# Runx1 and Runx2 inhibit fibrotic conversion of cellular niches for hematopoietic stem cells

**DOI:** 10.1038/s41467-022-30266-y

**Published:** 2022-05-12

**Authors:** Yoshiki Omatsu, Shota Aiba, Tomonori Maeta, Kei Higaki, Kazunari Aoki, Hitomi Watanabe, Gen Kondoh, Riko Nishimura, Shu Takeda, Ung-il Chung, Takashi Nagasawa

**Affiliations:** 1 Laboratory of Stem Cell Biology and Developmental Immunology, Graduate School of Frontier Biosciences, Osaka University, Suita, Osaka 565-0871 Japan; 2 Laboratory of Stem Cell Biology and Developmental Immunology, Graduate School of Medicine, Osaka University, Suita, Osaka 565-0871 Japan; 3 Laboratory of Stem Cell Biology and Developmental Immunology, WPI Immunology Frontier Research Center, Osaka University, Suita, Osaka 565-0871 Japan; 4 Laboratory of Stem Cell Genetics, Institute for Frontier Life and Medical Sciences, Kyoto University, Sakyo-ku, Kyoto, 606-8507 Japan; 5 Laboratory of Animal Experiments for Regeneration, Institute for Frontier Life and Medical Sciences, Kyoto University, Sakyo-ku, Kyoto, 606-8507 Japan; 6 Department of Molecular and Cellular Biochemistry, Graduate School of Dentistry, Osaka University, Suita, Osaka 565-0871 Japan; 7 Endocrinology Division, Toranomon Hospital, Minato-ku, Tokyo, 105-8470 Japan; 8 Department of Bioengineering, Graduate School of Engineering, The University of Tokyo, Bunkyo-ku, Tokyo, 113-8656 Japan

**Keywords:** Stem-cell niche, Haematopoietic stem cells, Mesenchymal stem cells, Stem-cell research, Bone

## Abstract

In bone marrow, special microenvironments, known as niches, are essential for the maintenance of hematopoietic stem cells (HSCs). A population of mesenchymal stem cells, termed CXC chemokine ligand 12 (CXCL12)-abundant reticular (CAR) cells or leptin receptor-expressing cells are the major cellular component of HSC niches. The molecular regulation of HSC niche properties is not fully understood. The role of Runx transcription factors, Runx1 and Runx2 in HSC cellular niches remains unclear. Here we show that Runx1 is predominantly expressed in CAR cells and that mice lacking both Runx1 and Runx2 in CAR cells display an increase in fibrosis and bone formation with markedly reduced hematopoietic stem and progenitor cells in bone marrow. In vitro, Runx1 is induced by the transcription factor Foxc1 and decreases fibrotic gene expression in CAR cells. Thus, HSC cellular niches require Runx1 or Runx2 to prevent their fibrotic conversion and maintain HSCs and hematopoiesis in adults.

## Abstract (web summary)

The transcription factors, Runx1 and Runx2 are critical embryonically for generation of HSCs and osteoblasts, respectively. Here the authors show that adult mice lacking Runx1 and Runx2 in HSC-supporting CAR cells displayed an increase in fibrosis with reduced HSCs in bone marrow.

## Introduction

In the adult bone marrow, special microenvironments, known as niches, are essential for the maintenance of hematopoietic stem cells (HSCs) that sustain hematopoiesis and the production of blood cells, including immune cells^1–3^. HSCs are in contact with their niches that provide critical signals. The identity of HSC niches has been a subject of longstanding debate, but recent studies have demonstrated that a population of self-renewing mesenchymal stem cells, termed CXC chemokine ligand 12 (CXCL12)-abundant reticular (CAR) cells, which overlap strongly with leptin receptor-expressing (LepR)^+^ cells, are the major cellular component of HSC niches in the bone marrow, giving rise to adipocytes and osteoblasts^4–12^. However, the molecular regulation of CAR/LepR^+^ cell development and properties is not fully understood.

CAR cells are the major producer of hematopoietic cytokines, including CXCL12 and stem cell factor (SCF), which are critical for the maintenance of hematopoietic stem and progenitor cells (HSPCs)^6,8,10,13,14^. In addition, the transcription factors, Foxc1 and Ebf1/3 are specifically expressed in CAR cells and play a critical role in the formation and maintenance of HSC niches, inhibiting adipocyte and osteoblast differentiation of CAR cells, respectively^9,15^. In contrast, CAR cells have much lower levels of fibrotic gene expression compared with fibrogenic cells in primary myelofibrosis^16^; however, the transcription factors involved in this remain unclear.

We have previously shown that CAR cells express high levels of the Runx transcription factor *Runx2* (ref. ^8^), prompting us to investigate the role of Runx transcription factors in formation and maintenance of HSC niches. Members of the Runx transcription factors share a highly conserved DNA binding region with the Drosophila pair-rule gene runt, which has the ability to make contact with the same DNA motif. Runx1 is known to be an important regulator of HSPCs. In mouse embryos, Runx1 is expressed in hemogenic endothelial cells and mice lacking Runx1 have no HSCs, indicating that Runx1 is essential for the establishment of definitive hematopoiesis in hemogenic endothelial cells^17,18^. In adults, it has been shown that loss of Runx1 leads to an increase in HSCs, myeloid progenitors with upregulation of stem cell and megakaryocytic transcription programs, and aberrant megakaryocyte progenitors and a decrease in B cell progenitors and megakaryocytes in the bone marrow as well as T cell progenitors in the thymus^19–21^. Clinically, Runx1 is one of the most frequently mutated genes in hematopoietic cells in hematological malignancies^22^. Somatic mutations and chromosomal rearrangements involving Runx1 are frequently observed in abnormal hematopoietic cells in myelodysplastic syndrome (MDS), acute myeloid leukemia (AML), acute lymphoblastic leukemia (ALL), and chronic myelomonocytic leukemia (CMML) patients^22^. On the other hand, the expression of Runx2 occurs at sites of bone formation early during skeletal development^23–25^. Embryos deficient for Runx2 have an almost perfectly patterned skeleton composed entirely of cartilage but lack bones^24,25^, indicating that Runx2 is essential for generation of osteoblasts. In adults, loss of Runx2 in hematopoietic cells leads to a decrease in plasmacytoid dendritic cells that mediate type I interferon responses to viral infection^26^.

Here, we have shown that Runx1 is highly and predominantly expressed in CAR cells and that mice lacking both Runx1 and Runx2 in CAR cells exhibit a marked reduction in HSPCs and increased bone formation and fibrosis in the bone marrow. These findings indicate that hemogenic Runx1 or osteogenic Runx2 inhibits fibrotic conversion of marrow mesenchymal stem cells and maintain HSC niches.

## Results

### *Runx1* is predominantly expressed in CAR cells in the marrow

**Fig. 1:**
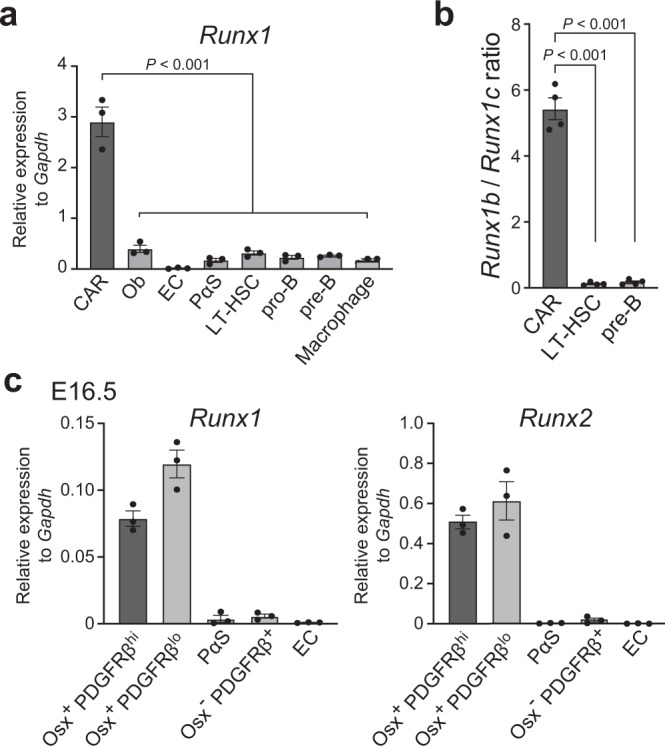
*Runx1* is predominantly expressed in CAR cells in adult bone marrow. **a** Relative mRNA expression levels of *Runx1* in CAR cells, osteoblasts (Ob), endothelial cells (EC), PαS cells, LT-HSCs, pro-B cells, pre-B cells, and F4/80^+^ macrophages in the bone marrow of 15-week-old *CXCL12-GFP* mice (*n* = 3 mice per group). **b** The ratio of *Runx1b* mRNA levels to *Runx1c* mRNA levels in CAR cells, LT-HSCs, and pre-B cells (*n* = 4 mice per group). **c** Relative mRNA expression levels of *Runx1* and *Runx2* in Osx-GFP^+^PDGFRβ^hi^ CAR cell progenitors, Osx-GFP^+^PDGFR-β^lo^ osteoblast progenitors, PαS cells, Osx-GFP^-^PDGFRβ^+^ non-endothelial cells, and EC in E16.5 *Osx-GFP* mice (*n* = 3 mice per group). All error bars represent SD of the mean. Statistical significances were calculated using one-way ANOVA with Dunnett’s test (**a**, **b**). Source data are provided as a Source Data file.

We analyzed relative mRNA expressions of *Runx1* in adult bone marrow cell populations. Quantitative real-time polymerase chain reaction with reverse transcription (qRT-PCR) analysis of 15-week-old CXCL12-GFP mice showed that expression levels of *Runx1* mRNA in CXCL12-GFP^hi^ CAR cells were much higher than those in other bone marrow nonhematopoietic cell populations, including alkaline phosphatase (ALP)^hi^CXCL12-GFP^lo^ osteoblasts, Sca-1^+^CD31^+^ endothelial cells, and platelet-derived growth factor receptor α (PDGFRα)^+^Sca-1^+^CD45^-^Ter119^-^ (PαS) cells^27^, as well as hematopoietic cell populations, including CD34^-^CD150^+^CD48^-^Lin^-^Sca-1^+^c-kit^+^ cells, which are highly enriched for long-term repopulating HSCs (LT-HSCs), c-kit^+^CD19^+^IgM^-^ pro-B cells, c-kit^-^CD19^+^IgM^-^ pre-B cells, and F4/80^+^ macrophages (Fig. 1a). Transcription of the Runx1 gene is under the control of two alternative promoters, which generate distal and proximal *Runx1* transcripts that produce Runx1c and Runx1b protein isoforms, respectively^28^. By qRT-PCR, we found that *Runx1b* mRNA was dominant in CAR cells whereas *Runx1c* was dominant in hematopoietic cells, including LT-HSCs and pre-B cells (Fig. 1b). We next analyzed relative mRNA expressions of *Runx1* as well as *Runx2*, which was shown to be abundantly expressed in CAR cells^8^, in fetal bone marrow cell populations. qRT-PCR analysis of embryonic day (E) 16.5 transgenic mice expressing the green fluorescent protein (GFP) reporter gene under the control of Osterix (*Osx/Sp7*) regulatory elements (*Osx-GFP*) mice showed that expression levels of *Runx1* and *Runx2* in CAR cell progenitors^15^ were similar to those in Osx-GFP^+^ osteoblast progenitors but higher than those in other nonhematopoietic cell populations (Fig. 1c).

### HSC niches are formed in mice lacking Runx1 or Runx2

**Fig. 2:**
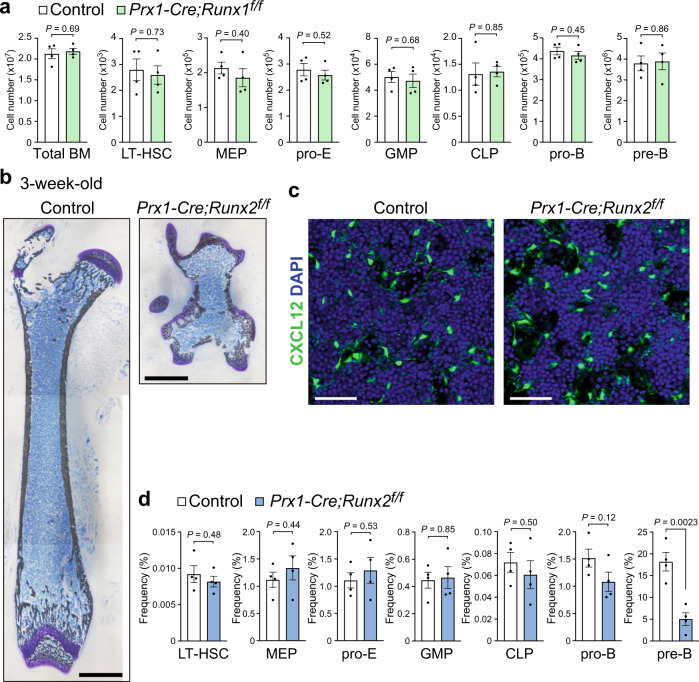
HSC niches are formed in mice lacking Runx1 or Runx2. **a**–**d** Bone marrow from 3-week-old control, *Prx1-Cre;Runx1*^*f/f*^ (**a**) or *Prx1-Cre;Runx2*^*f/f*^*;CXCL12-GFP* mice (**b**, **c**, **d**) was analyzed. **a** Total hematopoietic cell counts and the numbers of LT-HSCs, MEPs, proerythroblasts (pro-E), GMPs, CLPs, pro-B cells, and pre-B cells (*n* = 4 mice per group). **b** von Kossa and toluidine blue staining of femurs. Bars: 1 mm. **c** Confocal microscopy images showing expression of CXCL12-GFP. Bars: 100 μm. **d** Frequencies of LT-HSCs, MEPs, pro-E, GMPs, CLPs, pro-B cells, and pre-B cells (*n* = 4 mice per group). All error bars represent SD of the mean. Statistical significances were calculated using the two-tailed unpaired Student’s *t*-test. Source data are provided as a Source Data file.

To examine the role of Runx1 or Runx2 in CAR cell development, we analyzed mice with conditional floxed *Runx1* or *Runx2* alleles (*Runx1*^*f/f*^ or *Runx2*^*f/f*^ mice) in conjunction with transgenic mice expressing Cre recombinase under the control of *Prx1* regulatory elements (*Prx1-Cre* mice)^29^, in which Runx1 or Runx2 is deleted in all mesenchymal cells in developing limbs (*Prx1-Cre;Runx1*^*f/f*^ or *Prx1-Cre;Runx2*^*f/f*^ mice). *Prx1-Cre;Runx1*^*f/f*^ mice were viable^30^ and showed normal development of bone, bone marrow, and CAR cells at 3 weeks of age (Fig. 2a and Supplementary Fig. 1). On the other hand, *Prx1-Cre;Runx2*^*f/f*^ mice were born and viable but exhibited a severe reduction in the length of all limbs compared to control animals at 3 weeks of age. Histological analysis of femurs from *Prx1-Cre;Runx2*^*f/f*^ mice crossed to mice with GFP reporter gene knocked into the *CXCL12* locus (*CXCL12-GFP* mice) showed that there existed bone-like structures, which appear as woven bone and surround the bone marrow cavity (Fig. 2b). Although bone marrow cavity was reduced in length, CXCL12-GFP^+^ CAR cells, which exhibited a normal morphology with long processes, were present and their density appeared to be normal in the marrow cavity (Fig. 2c). Bone marrow volume and the total hematopoietic cell counts were reduced in the mutant marrow. However, by flow cytometry, there were no significant differences in frequencies of LT-HSCs, Lin^-^Sca-1^-^c-kit^+^CD34^-^FcγRII/III^lo^ megakaryocyte/erythrocyte progenitors (MEPs), c-kit^+^CD71^+^Ter119^lo^ proerythroblasts, Lin^-^Sca-1^-^c-kit^+^CD34^+^FcγRII/III^hi^ granulocyte/macrophage progenitors (GMPs), Lin^-^IL-7Rα^+^Flt3^+^ common lymphoid progenitors (CLPs), or pro-B cells between mutants and control animals. The frequencies of pre-B cells were only slightly reduced in the mutants (Fig. 2d). By qRT-PCR, CAR cells from the mutants had relatively normal expression of *Runx1*, platelet-derived growth factor receptor β (*PDGFRβ*), *LepR*, *CXCL12*, *SCF* (*Kitl*) *Foxc1*, *Ebf1*, and *Ebf3* (Supplementary Fig. 2). They had slightly reduced expression of Interleukin-7 (*IL-7*) (ref. ^13^) and much lower expression of *Osx* in CAR cells compared with control animals (Supplementary Fig. 2). Mice lacking Osx, genetically downstream of Runx2 (ref. ^31^), (*Prx1-Cre;Osx*^*f/f*^ mice) showed similar phenotypes to Runx2 mutants (Supplementary Fig. 3).

### HSC niches are maintained in mice lacking Runx1 or Runx2

To examine the role of Runx1 or Runx2 in CAR cell maintenance, we next crossed *Runx1*^*f/f*^ or *Runx2*^*f/f*^ mice with mice expressing the CreERT2 transgene under the control of the endogenous *Ebf3* locus, in which Cre recombinase can be activated in CAR cells but not in other bone marrow hematopoietic or nonhematopoietic populations upon tamoxifen treatment (*Ebf3-CreERT2* mice)^9^. *Ebf3-CreERT2;Runx1*^*f/f*^ or *Ebf3-CreERT2;Runx2*^*f/f*^ mice were subjected to a tamoxifen pulse for a week beginning at 10 weeks of age and analyzed 10–14 weeks after tamoxifen treatment. Flow cytometric analysis showed that the frequencies of LT-HSCs and the numbers of LT-HSCs, MEPs, c-kit^+^CD71^+^Ter119^lo^ proerythroblasts, GMPs, and CLPs in the bone marrow from tamoxifen-treated *Ebf3-CreERT2;Runx1*^*f/f*^ or *Ebf3-CreERT2;Runx2*^*f/f*^ mice were similar to those in control animals (Supplementary Fig. 4a, d).The numbers of pro-B cells and pre-B cells were unaltered in tamoxifen-treated *Ebf3-CreERT2;Runx1*^*f/f*^ mice (Supplementary Fig. 4a) but slightly reduced in tamoxifen-treated *Ebf3-CreERT2;Runx2*^*f/f*^ mice compared with control animals (Supplementary Fig. 4d). qRT-PCR analysis of the mutants showed that CAR cells had relatively normal expression of *PDGFRβ*, *LepR*, *CXCL12*, *SCF*, *Ebf1*, *Ebf3*, which are preferentially expressed in CAR cells, as well as fibrotic genes, including type I collagen α1 (*Col1a1*), type III collagen α1 (*Col3a1*), and type VI collagen α3 (*Col6a3*), and reduced expression of *Osx* (Supplementary Fig. 4b, c, e, f). Expression of *Foxc1* and *IL-7* was unaltered in tamoxifen-treated *Ebf3-CreERT2;Runx1*^*f/f*^ mice (Supplementary Fig. 4b) but slightly reduced in tamoxifen-treated *Ebf3-CreERT2;Runx2*^*f/f*^ mice (Supplementary Fig. 4e). Mice lacking Osx (*Ebf3-CreERT2;Osx*^*f/f*^ mice) showed similar phenotypes to Runx1 mutants (Supplementary Fig. 5).

### Mice lacking Runx1 and Runx2 in CAR cells have reduced HSPCs

**Fig. 3:**
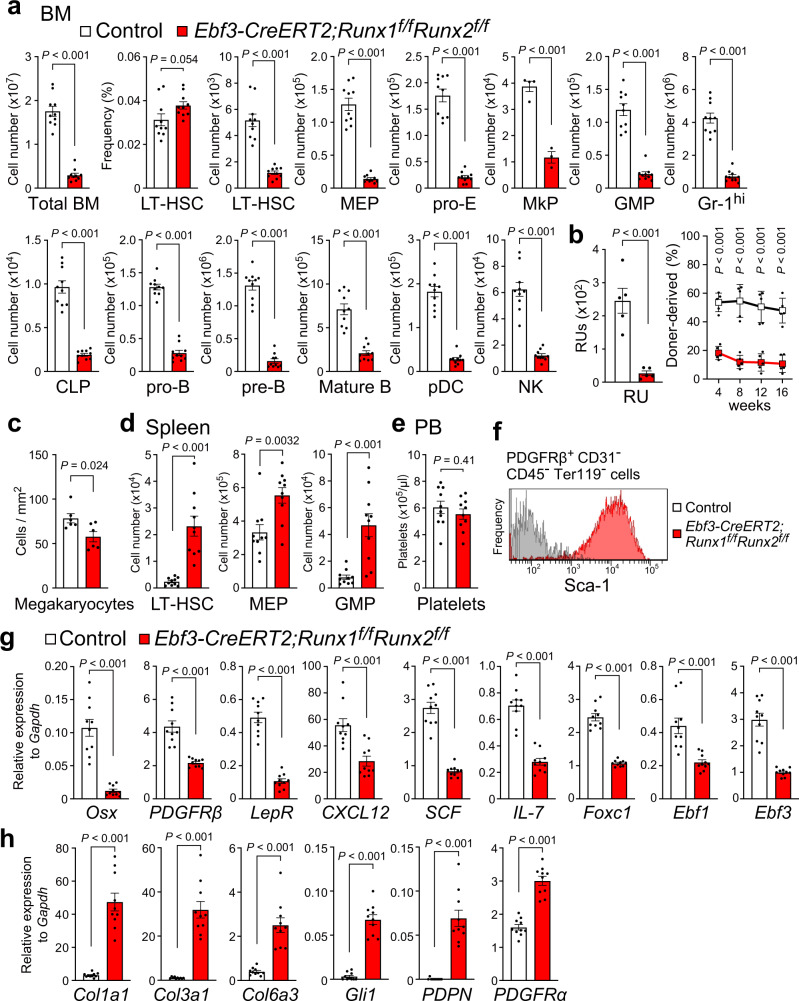
Markedly reduced HSPCs and impaired HSC niches in mice lacking both Runx1 and Runx2 in CAR cells. **a**–**h** Femurs and tibias (**a**–**c**, **f**–**h**), spleen (**d**), and peripheral blood (**e**) from 20- to 24-week-old tamoxifen-treated control and *Ebf3-CreERT2;Runx1*^*f/f*^*Runx2*^*f/f*^ mice were analyzed. **a** Total hematopoietic cell counts, frequencies of LT-HSCs, and the numbers of LT-HSCs, MEPs, pro-E, MkPs, GMPs, Gr-1^hi^ granulocytes, CLPs, pro-B cells, pre-B cells, mature B cells, pDCs, and NK cells (*n* = 10 mice per group). **b** The numbers of functional HSCs were estimated using repopulating units (RUs) (*n* = 5 mice per group). **c** The numbers of megakaryocytes per high powered field from bone marrow (*n* = 6 mice per group). **d** The numbers of LT-HSCs, MEPs, and GMPs in spleen (*n* = 10 mice per group). **e** Platelet counts in peripheral blood (PB) (*n* = 10 mice per group). **f** Cell surface expression of Sca-1 in CD31^-^CD45^-^Ter119^-^PDGFRβ^+^ CAR cells. **g**, **h** Relative mRNA expression levels of *Osx*, *PDGFRβ*, *LepR*, *CXCL12*, *SCF*, *IL-7*, *Foxc1*, *Ebf1*, *Ebf3* (**g**), *Col1a1*, *Col3a1*, *Col6a3*, *Gli1*, *PDPN*, and *PDGFRα* (**h**) in sorted CAR cells (*n* = 10 mice per group). All error bars represent SD of the mean. Statistical significances were calculated using the two-tailed unpaired Student’s *t*-test. Source data are provided as a Source Data file.

Since the expression of *Runx2* or *Runx1* was unaltered in CAR cells in the bone marrow from tamoxifen-treated *Ebf3-CreERT2;Runx1*^*f/f*^ or *Ebf3-CreERT2;Runx2*^*f/f*^ mice, respectively (Supplementary Fig. 4b, e), we analyze mice, in which both Runx1 and Runx2 were deleted from CAR cells. *Prx1-Cre;Runx1*^*f/f*^*Runx2*^*f/f*^ mice die perinatally with protruded internal organs due to the absence of sternum in contrast to the normal sternal fusion in *Prx1-Cre;Runx2*^*f/f*^ mice^30^. Histological analysis showed that femurs from *Prx1-Cre;Runx1*^*f/f*^*Runx2*^*f/f*^ mice lacked bone and bone marrow like *Prx1-Cre;Runx2*^*f/f*^ mice at E18.5 (ref. ^30^). We, therefore, generated *Ebf3-CreERT2;Runx1*^*f/f*^*Runx2*^*f/f*^ mice, in which both Runx1 and Runx2 were deleted from CAR cells upon tamoxifen treatment. These mice were subjected to a tamoxifen pulse for a week beginning at 10 weeks of age and analyzed 10–14 weeks after tamoxifen treatment. Flow cytometric analysis showed that the frequencies of LT-HSCs were unaltered but the total hematopoietic cell counts and numbers of phenotypic LT-HSCs, MEPs, proerythroblasts, Lin^-^Sca-1^-^c-kit^+^CD150^+^CD41^+^ megakaryocyte-committed progenitors (MkPs)^32^, GMPs, Gr-1^hi^CD11b^+^ granulocytes, CLPs, pro-B, pre-B cells, B220^hi^IgD^+^ mature B cells, plasmacytoid dendritic cells (pDCs), and NK cells were markedly reduced in the bone marrow of mutants compared with control animals (Fig. 3a). Additionally, we estimated the numbers of functional HSCs using repopulating units (RU), based on a competitive repopulation assay and found that RUs were markedly reduced in the mutant bone marrow (Fig. 3b). Histological analysis showed that numbers of morphologically identifiable CD41^+^CD31^+^ megakaryocytes per high powered field from bone marrow were modestly reduced in the mutants (Fig. 3c). Furthermore, the numbers of phenotypic LT-HSCs, MEPs, and GMPs were increased in the spleen of the mutants (Fig. 3d), suggesting that impaired hematopoiesis in the bone marrow induced extramedullary hematopoiesis. Peripheral blood (PB) platelet counts were unaltered in the mutants (Fig. 3e).

Flow cytometric analysis showed that CAR cells had increased cell surface expression of Sca-1 in the bone marrow of tamoxifen-treated *Ebf3-CreERT2;Runx1*^*f/f*^*Runx2*^*f/f*^ mice (Fig. 3f). qRT-PCR analysis showed that CAR cells from the mutants displayed reduced expression of *Osx*, *PDGFRβ*, *LepR*, and key HSC niche factors, including *CXCL12*, *SCF*, *IL-7*, *Foxc1*, *Ebf1*, and *Ebf3*, compared with control animals (Fig. 3g). Of note, some mutants had a modest reduction in *CXCL12*, *SCF*, and *Ebf1* expression in CAR cells but had a marked reduction in HSC number and CAR cells from the mutants displayed markedly increased expression of fibrotic genes, including *Col1a1*, *Col3a1*, *Col6a3*, and *Gli1* (ref. ^33^) as well as podoplanin (*PDPN*), which is highly expressed in fibroblastic reticular cells in lymph nodes^34^, and slightly increased expression of *PDGFRα*, which is required for bone marrow fibrosis^16^ (Fig. 3h). These results indicate that CAR cells undergo fibrotic differentiation and their HSPC niche function is impaired in the absence of Runx1 and Runx2.

### Mice lacking Runx1 and Runx2 in CAR cells have increased fibrosis in the marrow

**Fig. 4:**
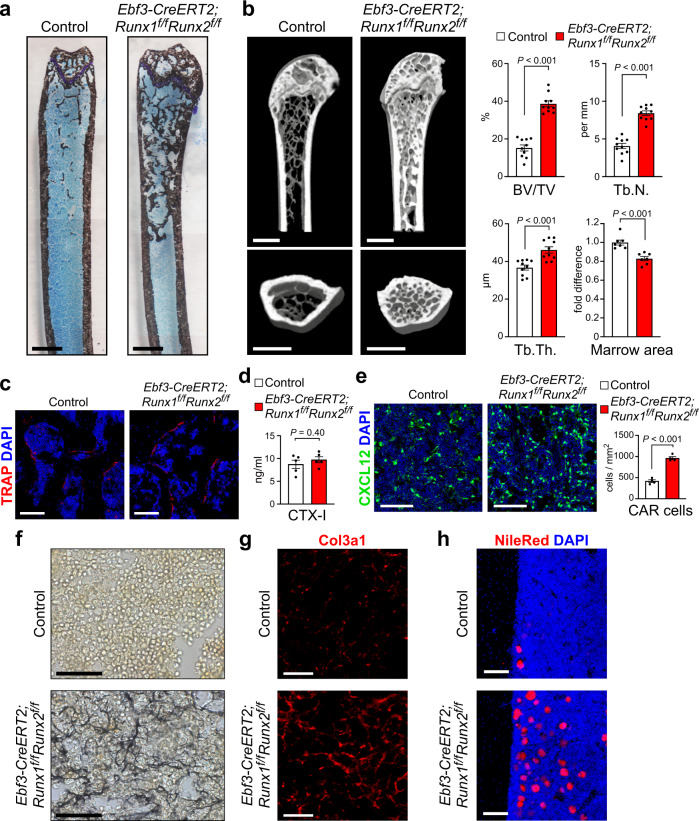
Increased bone formation and fibrosis in mice lacking Runx1 and Runx2 in CAR cells. **a**–**h** Bone marrow (**a**–**c**, **e**–**h**) and serum (**d**) from 20- to 24-week-old tamoxifen-treated control and *Ebf3-CreERT2;Runx1*^*f/f*^*Runx2*^*f/f*^*;CXCL12-GFP* mice were analyzed. **a** von Kossa and toluidine blue staining of section from the femur. Bars: 1 mm. **b** Three-dimensional μCT image of proximal femurs and analysis of the μCT image for bone volume per tissue volume (BV/TV) (*n* = 10 mice per group), trabecular number (Tb. N) (*n* = 10 mice per group), trabecular thickness (Tb. Th) (*n* = 10 mice per group), and bone marrow area (*n* = 7 mice per group). Bars: 1 mm. **c** TRAP^+^ osteoclasts in the metaphysis of the femur. Bars: 100 μm. **d** Serum CTX-I (*n* = 5 mice per group). **e** Confocal microscopy images showing expression of CXCL12-GFP and the frequencies of CAR cells (*n* = 4 mice per group). Bars: 100 μm. **f**, **g** Silver staining (**f**) and immunostaining with antibodies against Col3a1 (**g**) of sections from the femur. Bars: 50 μm (**f**) and 20 μm (**g**). **h** Imaging of femurs showing adipocytes, as assessed by Nile Red staining. Bars: 100 μm. All error bars represent SD of the mean. Statistical significances were calculated using the two-tailed unpaired Student’s *t*-test. Source data are provided as a Source Data file.

Although embryos lacking Runx2 have no bones, histological analysis of the bone marrow from tamoxifen-treated *Ebf3-CreERT2;Runx1*^*f/f*^*Runx2*^*f/f*^ mice showed that the trabecular bone numbers in the metaphysis were increased without any cartilaginous structure compared with control animals (Fig. 4a). Micro-computed tomography (μCT) analyses confirmed that the trabecular bone mass was increased and marrow area was slightly reduced in the long bones of the mutants (Fig. 4b). The numbers of cells lining the bone surface and expressing an osteoclast marker tartrate-resistant acid phosphatase (TRAP) were unaltered in the marrow sections of the mutants (Fig. 4c). Additionally, serum levels of bone resorption marker carboxy-terminal telopeptides of type I collagen (CTX-I), which reflect the whole-body amount of cleaved type I collagen by osteoclasts, were unaltered in the mutants (Fig. 4d). These results suggest that increased bone formation in the mutants does not result from defects in osteoclasts.

Histological analysis of the bone marrow from tamoxifen-treated *Ebf3-CreERT2;Runx1*^*f/f*^*Runx2*^*f/f*^*;CXCL12-GFP* mice showed that CXCL12-GFP^hi^ cells, which exhibited a CAR cell-like morphology, were present and their numbers were increased about 2-fold compared with those of CXCL12-GFP^hi^ CAR cells in control animals (Fig. 4e). The protein expression of CXCL12 and SCF was relatively normal in the mutants, presumably because of a decrease in their mRNA expression in CAR cells and an increase in the numbers of CAR cells (Supplementary Fig. 6). Importantly, in consistent with the increased expression of fibrotic genes in CAR cells lacking Runx1 and Runx2, silver staining showed massive fibrosis in bone marrow sections from the mutants whereas no significant reticulin deposition was detected in control animals (Fig. 4f). In addition, immunohistochemical analysis with antibodies against Col3a1 revealed that CXCL12-GFP^hi^ CAR cells and CXCL12-GFP^-^ cells expressing high levels of Col3a1 protein were almost absent in control animals but were markedly increased in the mutants (Fig. 4g). These fibrotic phenotypes were not observed in Runx1- or Runx2-deficient mice. Histological analysis showed that the numbers of adipocytes were increased in the bone marrow of the mutants (Fig. 4h).

### Runx1 or Runx2 decreased fibrotic gene expression in vitro

**Fig. 5:**
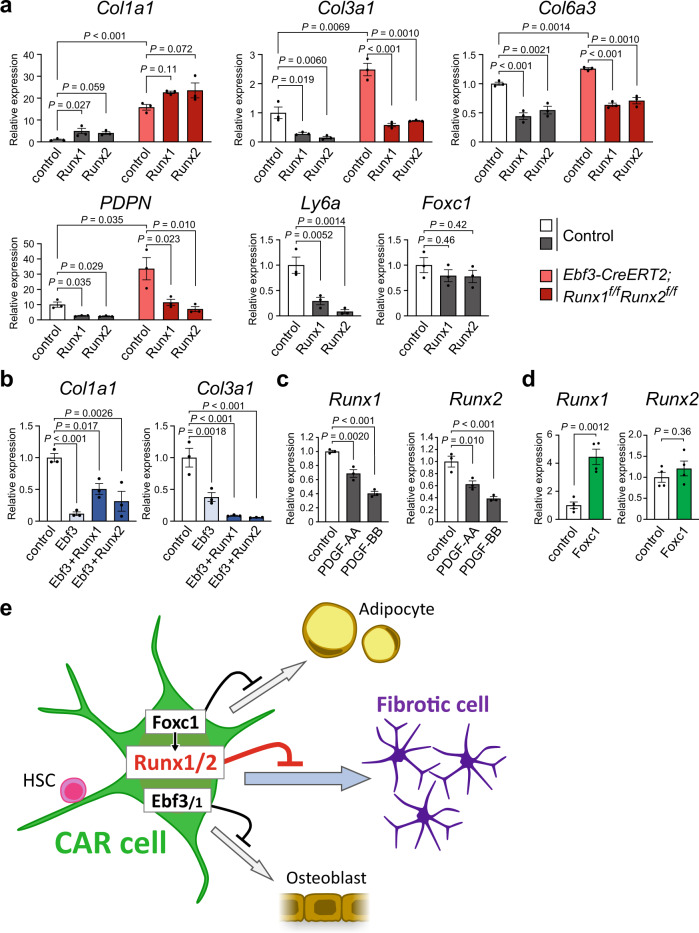
In vitro activities of Runx1 or Runx2 on fibrotic gene expression in CAR cells. **a** Analysis of CAR cells from control and tamoxifen-treated *Ebf3-CreERT2;Runx1*^*f/f*^*Runx2*^*f/f*^ mice transduced with retroviruses expressing *Runx1* and *Runx2* as well as empty vector in vitro. Relative mRNA expression levels of *Col1a1*, *Col3a1*, *Col6a3*, *PDPN*, *Sca-1* (*Ly6a*) and *Foxc1* in infected CAR cells cultured for 4 days (*n* = 3 biological replicates per group). **b** Analysis of CAR cells from wild-type mice transduced with retroviruses expressing *Ebf3*, *Ebf3* and *Runx1*, or *Ebf3* and *Runx2* as well as empty vector in vitro. Relative mRNA expression levels of *Col3a1* and *Col6a3* in infected CAR cells cultured for 4 days (*n* = 3 biological replicates per group). **c** Effects of PDGFs on *Runx1* and *Runx2* expression in CAR cells in vitro. Relative mRNA expression levels of *Runx1* and *Runx2* in CAR cells cultured in the presence of PDGF-AA or PDGF-BB for 3 days (*n* = 3 biological replicates per group). **d** Analysis of CAR cells transduced with retroviruses expressing *Foxc1* and empty vector in vitro. Relative mRNA expression levels of *Runx1* and *Runx2* in infected CAR cells cultured for 4 days (*n* = 3 biological replicates per group). **e** Working model. CAR cells are specialized mesenchymal stem cells, which express the specific transcription factors, including Runx1/2 as well as Foxc1 and Ebf1/3. Runx1/2 prevents fibrotic conversion of CAR cells to maintain HSC niches. All error bars represent SD of the mean. Statistical significances were calculated using one-way ANOVA with Dunnett’s test (**a**–**c**) and the two-tailed unpaired Student’s *t*-test (**d**). Source data are provided as a Source Data file.

To examine the in vitro functions of Runx1 or Runx2 in fibrotic gene expression in CAR cells, we infected sorted CAR cells with retroviruses expressing *Runx1* or *Runx2*. The enforced expression of *Runx1* or *Runx2* in CAR cells increased mRNA expression of *Col1a1* and decreased mRNA expression of fibrotic genes, including *Col3a1*, *Col6a3*, and *PDPN* as well as *Sca-1* (*Ly6a*) but did not alter *Foxc1* expression based on qRT-PCR in the culture (Fig. 5a). We next infected sorted CAR cells with retroviruses expressing *Runx1* or *Runx2* in addition to *Ebf3*, which inhibits osteoblast differentiation of CAR cells^9^. The enforced expression of *Ebf3*, *Ebf3* and *Runx1*, or *Ebf3* and *Runx2* in CAR cells decreased the expression of *Col1a1* and *Col3a1* as assessed by qRT-PCR in the culture (Fig. 5b). Cultured CAR cells from tamoxifen-treated *Ebf3-CreERT2;Runx1*^*f/f*^*Runx2*^*f/f*^ mice had an increase in the expression of fibrotic genes than that of control CAR cells in the culture (Fig. 5a). The increase was modest presumably because of reduction in the expression of *Runx1* and *Runx2* in control CAR cells after in vitro culture. The enforced expression of *Runx1* or *Runx2* in mutant CAR cells reduced the expression of fibrotic genes to normal or lower levels in the culture (Fig. 5a). It has been previously shown that PDGFRα in CAR/LepR^+^ cells is involved in bone marrow fibrosis^16^. We found that PDGFs reduced the expression of *Runx1* and *Runx2* in CAR cells in culture (Fig. 5c).

We next analyze whether Foxc1, which is essential for formation and maintenance of HSC niches in CAR cells^15^, induce *Runx1* or *Runx2* expression in CAR cells. The enforced expression of *Foxc1* markedly increased mRNA expression of *Runx1* but not *Runx2*, based on qRT-PCR in the culture (Fig. 5d). Considering that Foxc1 but not Runx1 is predominantly expressed in CAR cell progenitors in E16.5 embryos (Fig. 1c)^15^, Foxc1 might enhance the expression of Runx1 in CAR cells during development.

### Runx1 and Runx2 are decreased in CAR cells in a PMF model

**Fig. 6:**
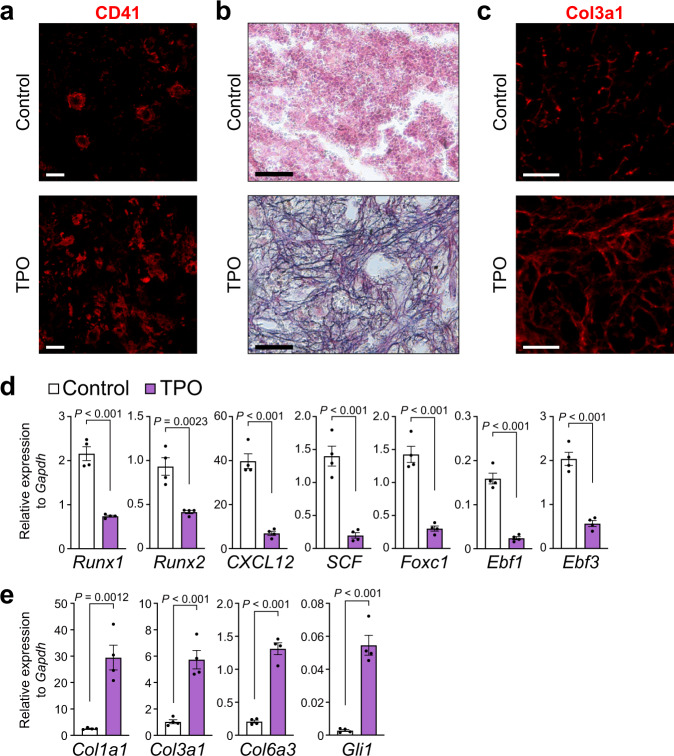
Expression of *Runx1* and *Runx2* is decreased in CAR cells in the PMF mouse model. **a**–**e** HSPCs infected with retroviruses expressing *TPO* were transplanted into irradiated mice (TPO). Recipients developing PMF-like disease were analyzed 8–9 weeks after transplantation. **a**–**c** Immunostaining with antibodies against CD41 (**a**) and Col3a1 (**c**) and silver staining (**b**) of sections from the femur. Bars: 50 μm (**a**, **c**) and 20 μm (**b**). **d**, **e** Relative mRNA expression levels of *Runx1*, *Runx2*, *CXCL12*, *SCF*, *Foxc1*, *Ebf1*, *Ebf3* (**d**), *Col1a1*, *Col3a1*, *Col6a3*, and *Gli1* (**e**) in sorted CAR cells (*n* = 4 mice per group). All error bars represent SD of the mean (**d**, **e**). Statistical significance was calculated using the two-tailed unpaired Student’s *t*-test. Source data are provided as a Source Data file.

Primary myelofibrosis (PMF) patients demonstrate myelofibrosis during the course of their disease^35^. We thus examine expression levels of *Runx1* and *Runx2* in CAR cells in PMF, using a PMF mouse model where HSPCs infected with retroviruses expressing thrombopoietin (*TPO*) were transplanted into irradiated mice^16^. Recipients of *TPO* transduced HSPCs developed PMF-like disease 8–9 weeks after transplantation. Immunohistochemical analysis with antibodies against CD41 revealed that megakaryocyte lineage cells were increased (Fig. 6a). Silver staining showed massive fibrosis in bone marrow sections from PMF mice, whereas no significant reticulin deposition was detected in control animals (Fig. 6b). Immunohistochemical analysis with antibodies against Col3a1 revealed that cells expressing high levels of Col3a1 protein were almost absent in control animals but were markedly increased in PMF mice (Fig. 6c). qRT-PCR analysis showed that CAR cells from PMF mice displayed reduced expression of *Runx1* and *Runx2* as well as key HSC niche factors, including *CXCL12*, *SCF*, *Foxc1*, *Ebf1*, and *Ebf3*, compared with control animals (Fig. 6d). In consistent with massive fibrosis in bone marrow sections, CAR cells displayed markedly increased expression of fibrotic genes, including *Col1a1*, *Col3a1*, *Col6a3*, and *Gli1* in PMF mice (Fig. 6e).

## Discussion

We show that Runx1 and Runx2 are highly expressed in CAR/LepR^+^ cells in the bone marrow. Mice lacking Runx2 failed to generate osteoblasts and showed delayed invasion of blood vessels into hypertrophic cartilage and bone marrow formation probably due to defective development of chondrocytes during endochondral bone development^36–38^. However, we show that CAR cells were largely unaltered in the absence of Runx2 (ref. ^39^) or Osterix, which is genetically downstream of Runx2. Considering that osteoblasts and primordial CAR cells are generated from heterogeneous perichondrial cells during embryogenesis^15,40^, these results indicate that Runx2 is essential for the development of osteoblasts in the fetus but not for the development and maintenance of CAR cells.

In contrast, CAR cell-specific deletion of both Runx1 and Runx2 induced massive fibrosis with increased expression of fibrotic genes in CAR cells in the bone marrow. Consistent with this, enforced expression of *Runx1* or *Runx2* decreased the expression of fibrotic genes, including *Col3a1* and *Col6a3*, in CAR cells in vitro. Runx1/2 overexpression increased expression of a fibrotic gene *Col1a1* in cultured CAR cells, in which *Ebf3* expression was much lower than that in bone marrow CAR cells; however, enforced expression of *Ebf3* blocked this effect and reduced the expression of *Col1a1* in cultured CAR cells (Fig. 5b). Thus, in the absence of Runx1 and Runx2, *Col1a1* expression was increased presumably because of a decrease in *Ebf3* expression in mutant CAR cells in the marrow. Together, Runx1/2 are required for preventing fibrotic conversion of CAR cells and they would act in a cell-autonomous fashion (Fig. 5e).

The stromal cells responsible for fibrosis are thought to be activated by the abnormal megakaryocytes in myelofibrosis^41^. Previous studies have shown that genetic ablation of Runx1 in hematopoietic cells caused an increase in HSCs, GMPs with upregulation of stem cell and megakaryocytic transcription programs, and aberrant progenitors of megakaryocytes as well as a reduction in PB platelet counts, indicating that Runx1 in HSPCs is involved in megakaryocytic differentiation^19–21,42^. In contrast, megakaryocytes cell size appeared normal, frequencies of megakaryocytes were modestly reduced, and numbers of HSCs and MkPs were markedly reduced in tamoxifen-treated *Ebf3-CreERT2;Runx1*^*f/f*^*Runx2*^*f/f*^ mice. In addition, expression of *PDGF*s and *TGF-β1*, which are suggested to be produced by megakaryocytes and induce myelofibrosis in PMF mouse models^16,43^, in HSPCs, including MkPs, is unaltered in the mutants (Supplementary Fig. 7). Although we cannot entirely exclude the possibility that megakaryocytes are altered and affect CAR cells in the mutants, these results suggest that megakaryocytes and their progenitors are not responsible for myelofibrosis in the mutants, supporting the idea that effects of loss of function of Runx1/2 in CAR cells on the marrow fibrotic phenotype are cell autonomous.

Even after adjusting for bone marrow volume, the numbers of HSPCs and immune cells were markedly reduced in mice lacking Runx1 and Runx2 in CAR cells (Figs. 3a and 4b), indicating that Runx1/2 play a critical role in the maintenance of niches for HSCs and hematopoiesis. There is the possibility that increased fibrotic proteins, including Col1a1, Col3a1, and Col6a3, in CAR cells prevent CAR cells from providing critical signals to HSPCs and immune cells. Additionally, defective hematopoiesis in the absence of Runx1/2 would be partially attributed to decreased expression levels of *CXCL12*, *SCF*, *Foxc1*, *Ebf1*, and *Ebf3*, which are essential for the maintenance of HSPCs^6,9,15,44^. However, some *Ebf3-CreERT2;Runx1*^*f/f*^*Runx2*^*f/f*^ mice had a modest reduction in *CXCL12*, *SCF*, *Foxc1*, *Ebf1*, and *Ebf3* expression in CAR cells but had a marked increase in fibrotic gene expression and a marked reduction in HSC numbers. This result and the finding that CAR cell numbers were doubled in these mutants, which might be beneficial for HSCs, support the idea that defects in HSC niche functions other than reduced expression of HSC niche factors in CAR cells caused a marked reduction in HSC numbers in these mutants. How fibrotic conversion of CAR cells affects HSCs and hematopoiesis is an important question for the future.

Unexpectedly, mice lacking both Runx1 and Runx2 in adult CAR cells displayed an increase in bone formation as well as adiposity in the marrow. Considering that the majority of CAR cells remained undifferentiated in these mutants, this raises the possibility that Runx1/2 exert inhibitory effects on sensitivity to unknown extracellular and/or intracellular cue that stimulates osteoblast or adipocyte differentiation of CAR cells in adult bone marrow.

Foxc1 and Ebf1/3, which are specifically expressed in CAR cells and critical for their HSPC niche functions^9,15^, were reduced but detectable in CAR cells lacking both Runx1 and Runx2. However, enforced expression of *Runx1* did not increase expression of *Foxc1* and *Ebf3*. Considering that enforced expression of *Foxc1* enhanced expression of *Runx1*, Foxc1 might enhance the expression of *Runx1* in CAR cells (Fig. 5e). How expression of Runx1/2, Foxc1, and Ebf1/3 is enhanced in CAR cells is an important question for the future.

Expression levels of *Runx1* are much higher in CAR cells than those in osteoblasts, although expression levels of *Runx2* are similar between these cell populations. Moreover, HSPC niche functions of CAR cells were dramatically altered in the absence of both Runx1 and Runx2 but largely unaltered in the absence of Runx2, whereas fetal osteoblasts were depleted in the absence of Runx2. Thus, Runx1 compensates for the inactivation of Runx2 in CAR cells but not in fetal osteogenic progenitors.

Clinically, it is known that myelofibrosis is driven by mutations in HSPCs that alter the proliferation properties of the HSPCs. Our results raise the possibility that hematopoietic cells with the driver mutations found in myelofibrosis act on HSC cellular niches (CAR cells) to reduce their Runx1/2 expression, resulting in an increase in myelofibrosis. Consistent with this, CAR cells from PMF mice with myelofibrosis displayed reduced expression of *Runx1* and *Runx2* compared with control animals (Fig. 6c). In addition, PDGFs, which induce myelofibrosis in the PMF mouse model^16^, reduced expression of *Runx1* and *Runx2* in cultured CAR cells. We analyzed available data sets comparing gene expression in bone marrow cells from PMF patients with healthy donors^45^. Although expression of *RUNX1* and *RUNX2* was unaltered, expression of fibrotic genes, including *COL1A1*, *COL3A1*, *COL6A3*, and *GLI1* as well as *PDPN*, was also unaltered in bone marrow cells from PMF patients, suggesting that the proportion of fibrogenic cells and/or fibroblasts was not high in the cryopreserved cells from the bone marrow provided as samples from PMF patients^45^. Thus, it will be important to analyze expression of *RUNX1* and *RUNX2* in fibrogenic cells as well as CAR cells^46^ of PMF patients to understand pathogenesis of fibrosis; however, it is technically difficult to obtain informative bone marrow fibrogenic cells and fibroblasts from PMF patients. TGF-β signaling activated in marrow stromal cells has been hypothesized to drive the progression of Philadelphia translocation (Ph)-negative myeloproliferative neoplasms (MPN) to PMF^43,45^. If so, although it has been reported that TGF-β signaling pathways transcriptionally activate Runx proteins in tumor microenvironments^47^, our results raise the possibility that TGF-β signaling might inhibit the Runx1/2 expression and/or function in CAR cells to induce myelofibrosis. Thus, this study proposes a mechanism implicated in the pathogenesis of myelofibrosis; however, further studies are needed to address this issue.

On the other hand, *RUNX1* mutations are observed in the malignant stem cell clones of a minority of myelofibrotic patients, and only when these patients have progressed to AML^35^ and enforced expression of *Runx2* in HSPCs has been shown to induce AML in mouse^48^. In addition, *RUNX1* and/or *RUNX2* mutations in bone marrow nonhematopoietic cells, including CAR cells, have not been found in PMF patients. Thus, mutations or chromosomal rearrangements involving *Runx1* and/or *Runx2* would not contribute to progression of myelofibrosis.

For clinical application, our results raise the possibility that activators of CAR cell-specific RUNX1 can decrease bone marrow fibrosis, which might be applied to non-cell-autonomous therapies targeting the HSC niches, in hematological disorders, including MPN. In addition, Runx1 activators might enhance the formation of HSPC niches, which support ex vivo hematopoiesis, from non-niche cells, including dermal fibrogenic cells or induced pluripotent stem cell (iPSC)-derived fibrogenic cells in vitro. This study provides significant advances in our understanding of mesenchymal cells providing HSPC niches and bone.

## Methods

### Mice

Male and female mice were used between 3–24 weeks of age or at the age of embryonic day 16.5, depending on the respective experiments. C57BL/6 mice were purchased from Japan SLC, Inc. *Runx1*^*f/f*^ mice^30^, *Sp7*^*f/f*^ mice^37^, *Ebf3-CreERT2* knock-in mice^9^, *CXCL12-GFP* knock-in mice^49^, and *Osx-GFP* knock-in mice^50^ have been previously described. *Runx2*^*f/f*^ mice were generated with two loxP sites flanking exon 4 of *Runx2* by electroporation of a targeting vector into embryonic stem cells. These mice were backcrossed at least six times onto a C57BL/6 background. *Prx1-Cre* mice^29^ were obtained from The Jackson Laboratory. To induce CreERT2-mediated recombination, mice were injected i.p. with 2 mg tamoxifen (Sigma) four times every other day, and analyzed 10–14 weeks after the initial tamoxifen injection. Peripheral blood was measured on a hematology analyzer Celltac-α (Nihon Kohden). All mice were bred and maintained under specific pathogen-free conditions at the animal facilities of Osaka University. These mice were maintained in 12 h light/dark cycle, and the housing temperature and humidity were 23 ± 1.5 °C and 45 ± 15%, respectively. All animal experiments were performed in accordance with approved protocols of the Institutional Animal Care and Use Committees at Osaka University and Kyoto University.

### Antibodies

Monoclonal antibodies were purchased from BioLegend, BD Bioscience, eBioscience, or Miltenyi Biotec, unless otherwise noted: B220-PE/Cy5 (1:800, RA3-6B2, 15-0452-83, eBioscience), B220-PE (1:200, RA3-6B2, 103208, Biolegend), B220-PB (1:100, RA3-6B2, 103227, Biolegend), CD3ε-PE/Cy5 (1:400, 145-2C11, 100310, Biolegend), CD3ε-APC (1:100, 145-2C11, 100312, Biolegend), CD11b-PE/Cy5 (1:1600, M1/70, 101210, Biolegend), CD11b-PE/Cy7 (1:100, M1/70, 101216, Biolegend), CD19-PE/Cy5 (1:100, 1D3, 115510, Biolegend),CD19-PE (1:100, 1D3, 2016867, eBioscience), CD31-APC (1:200, MEC13.3, 102516, Biolegend), CD34-FITC (1:25, RAM34, 11-0341-85, eBioscience), CD41-FITC (1:100, MWReg30, 553848, BD Pharmingen), CD45-PE/Cy5 (1:800, 30-F11, 103110, Biolegend), CD45.1-FITC (1:100, A20, 110706, Biolegend), CD45.2-APCe780 (1:100, 104, 47-0454-82, eBioscience), CD48-PB (1:200, HM48-1, 103418, Biolegend), CD71-PE (1:400, C2, 553267, BD Pharmingen), CD150-BV421 (1:50, TC15-12F12.2, 115925, Biolegend), CD150-PE (1:100, TC15-12F12.2, 115904, Biolegend), c-Kit-APC (1:200, 2B8, 2078220, eBioscience), c-Kit-PE/Cy7 (1:200, 2B8, 105814, Biolegend), FcγRII/III-PE (1:100, 2.4G2, 553145, BD Pharmingen), Flt3-Biotin (1:100, A2F10, 135308, Biolegend), F4/80-Alexa 647 (1:500, BM8, 123122, Biolegend), Gr-1-PB (1:400, RB6-8C5, 108430, Biolegend), IgM-APC (1:100, II/41, 2056825, eBioscience), IgD-FITC (1:200, 11-26 c.2a, 405704, Biolegend), IL-7Rα-PE/Cy5 (1:50, A7R34, 15-1271-83, eBioscience), IL-7Rα-PE/Cy7 (1:100, A7R34, 135014, Biolegend), NK1.1-PE (1:100, PK136, 553165, BD Pharmingen), PDCA-1-FITC (1:50, JF05-1C2.4.1, 130-102-229, Miltenyi Biotec), Sca-1-PE/Cy7 (1:100, E13-161.7, 108114, Biolegend), Ter119-PE/Cy5 (1:400, Ter119, 116210, Biolegend), Ter119-APC (1:100, Ter119, 116212, Biolegend). The following polyclonal antibodies were used: PDGFRβ (1:200, R&D Systems, BAF1042), TRAP (1:100, Abcam, ab185716), and Col3a1 (1:100, Abcam, ab7778).

### Flow cytometric analysis and cell sorting

Bone marrow cells were isolated by flushing or crushing from femurs and tibias. Bone marrow nonhematopoietic cells were isolated by flushing or crushing from femurs, tibias, and humeri followed by enzymatic digestion with collagenase type I (Gibco) and DNase I (Sigma). Cells in bone fractions, including osteoblasts and PαS cells, were isolated by mechanical disruption and collagenase digestion of bones as described previously^8^. CAR cells were isolated as CXCL12-GFP^hi^Sca-1^-^CD31^-^CD45^-^Ter119^-^ cells from *CXCL12-GFP* mice or PDGFRβ^+^Sca-1^-^CD31^-^CD45^-^Ter119^-^ cells. Endothelial cells were isolated as CD31^+^Sca-1^+^CD45^-^Ter119^-^ cells. Osteoblasts were isolated as ALP^hi^CXCL12-GFP^lo^CD31^-^CD45^-^Ter119^-^ cells from *CXCL12-GFP* mice using ELF-97 phosphatase substrate (Invitrogen). All flow cytometric experiments and cell sorting were performed using a BD FACS Aria and FACS Diva 8.0.1 software (BD Biosystems). Gating strategies are included in Supplementary Fig. 8.

### Histology

Bone marrow sections were analyzed by immunofluorescence as described previously^10^. In brief, bone samples were fixed in 4% paraformaldehyde and equilibrated in 30% sucrose/phosphate-buffered saline (PBS). Fixed samples were embedded in OCT medium (Sakura) and frozen in cooled hexane. Sections (12 μm) of undecalcified femoral bone were generated by Kawamoto’s film method^51^ (Cryofilm transfer kit; Section-Lab). Sections were stained with von Kossa and toluidine blue. Reticulin staining was performed with ammoniacal silver solution (Muto Pure Chemicals) according to the manufacturer’s instructions. For immunohistochemistry, sections were first blocked with 5% FCS and then stained with antibodies in blocking buffer. Counts of CXCL12-GFP^+^ CAR cells were obtained from femur sections. Confocal microscopy was performed with an LSM 510 META (Carl Zeiss). Image analysis and cell quantification were performed using Zeiss ZEN 3.0 SR software (Carl Zeiss).

### Micro-computed tomography (μCT) analysis

The attached soft tissue in femurs was removed thoroughly and fixed in 4% paraformaldehyde. Micro-computed tomography scanning was performed using ScanXmate-RX Scanner (Comscantechno). Three-dimensional microstructural image data were reconstructed, and structural indices were calculated using TRI/3D-BON software (RATOC Systems).

### ELISA

As a bone resorption marker, serum CTX-I was measured with the RatLaps ELISA kit (Immunodiagnostic Systems) according to the manufacturer’s instructions. CXCL12 and SCF of humeri were measured as described previously^8^ using Quantikine ELISA Kit (R&D Systems) according to the manufacturer’s instructions.

### qRT-PCR

Relative mRNA expression was analyzed by qRT-PCR analysis performed with a Step One Plus (Applied Biosystems) using Thunderbird SYBR qPCR Mix (Toyobo). Total RNA was isolated from sorted cells using Isogen (Nippon Gene) and treated with DNase I (Invitrogen), and cDNA was synthesized using SuperScript VILO (Invitrogen) following the manufacturer’s instructions. Values for each gene were normalized to the relative quantity of *Gapdh* mRNA in each sample. The primers used for qRT-PCR are listed in Supplementary Table 1.

### Competitive repopulation assays

Competitive repopulation assays were performed using the CD45.1/CD45.2 congenic system. Unfractionated 1/20 of bone marrow cells (CD45.2) were transplanted into lethally irradiated (8 Gy) recipient mice (CD45.1) with 1×10^6^ competitor cells (CD45.1/CD45.2). Myeloid, B, and T cells in peripheral blood of the recipient mice were analyzed by flow cytometry using antibodies against Gr-1, B220, and CD3 for 16 weeks after transplantation. High turnover of myeloid cells provides a good measure of HSC activity, and repopulating units were calculated using Harrison’s formula as described previously^52^.

### Cell cultures and retroviral infections

*Runx1*, *Runx2*, *Ebf3*, and *Foxc1* cDNA were cloned into MSCV-based retroviral vectors. CAR cells from wild-type mice were isolated by flow cytometry, plated into 96-well plates (500-1,000 cells/well) in MF-start medium (Toyobo) supplemented with Y-27632 (Fujifilm Wako Pure Chemical) at 10 μM, and cultured with 5% O_2_ and 5% CO_2_ for 4–5 days. Retroviral transductions into cultured CAR cells were performed with plat-E cells as producers of viral supernatants as previously described^53^. After transduction, media were changed into MF-start medium. Four days after transduction, transduced (GFP^+^ and/or Kusabira Orange^+^) cells were sorted for qRT-PCR analysis.

For PDGF stimulation, after 4–5 days initial culture as described above, CAR cells were stimulated with recombinant mouse PDGF-AA or PDGF-BB (Fujifilm Wako Pure Chemical) at 250 ng/mL in DMEM supplemented with 5% FCS. Three days after stimulation, PI− cells were sorted for qRT-PCR analysis.

### PMF model mice

Mouse *TPO* cDNA was cloned into MSCV-based retroviral vectors. MSCV-IRES-GFP or MSCV-TPO-IRES-GFP was transiently transfected into Plat-E packaging cells to produce retroviruses^53^. Bone marrow CD48^-^Lin^-^Sca-1^+^c-kit^+^ cells were isolated from wild-type mice and cultured overnight in S-Clone SF-O3 media (Iwai North America) supplemented with mouse SCF (10 ng/ml, BioLegend), mouse TPO (20 ng/ml, R&D Systems), 100 μM 2-mercaptoethanol (Sigma-Aldrich), and 1% penicillin/streptomycin (Nacalai tesque). The pre-stimulated cells were infected with the retroviruses harboring MSCV-IRES-GFP or MSCV-TPO-IRES-GFP. The cells were harvested 48 h after the infection, and 6000-10,000 GFP^+^ cells were transplanted into lethally irradiated (8 Gy) recipient mice with 1 × 10^6^ competitor cells.

### Statistical analysis

One-way ANOVA with Dunnett’s multiple comparison test was used for all studies with more than two groups. For comparisons between two groups, unpaired two-tailed Student’s t-test was used. Statistical significances were calculated using GraphPad Prism 9.3.1 (GraphPad Software). All experiments were repeated at least three times with sufficient reproducibility.

### Reporting summary

Further information on research design is available in the Nature Research Reporting Summary linked to this article.

## Supplementary information

Supplementary Information

Reporting Summary

## Data Availability

Source data are provided with this paper.

## Source data

Source Data
