# Supplementary material for: Runx1 and Runx2 inhibit fibrotic conversion of cellular niches for hematopoietic stem cells
Source: Nat Commun. 2022 May 12;13:2654. doi: 10.1038/s41467-022-30266-y (PMC9098511; doi:10.1038/s41467-022-30266-y)
Supplement: Supplementary file 1 — Supplementary Information [file 41467_2022_30266_MOESM1_ESM.pdf]

**Supplemental Information.**

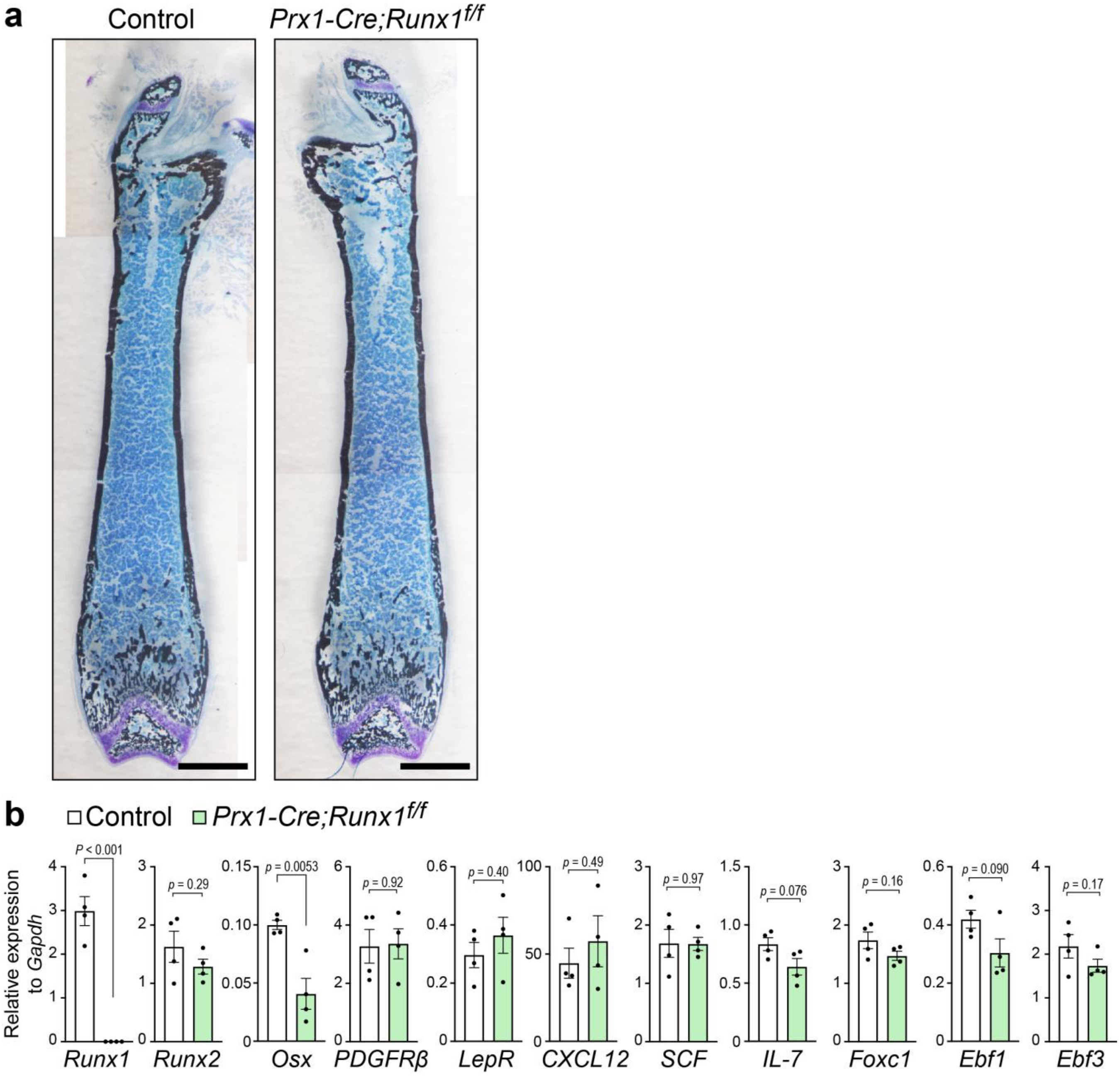


**Supplementary Figure 1. Runx1 is dispensable for the development of HSC niches**

**a**,**b** Bone marrow from 3-week-old control and *Prx1-Cre;Runx1$^{f/f}$* mice was analyzed.

**a** von Kossa and toluidine blue staining of femurs. Bars: 1 mm.

**b** Relative mRNA expression levels of *Runx1*, *Runx2*, *Osx*, *PDGFRβ*, *LepR*, *CXCL12*, *SCF*, *IL-7*, *Foxc1*, *Ebf1*, and *Ebf3* in sorted CAR cells (n = 4 mice per group).

All error bars represent SD of the mean. Statistical significances were calculated using the two-tailed unpaired Student's *t*-test. Source data are provided as a Source Data file.

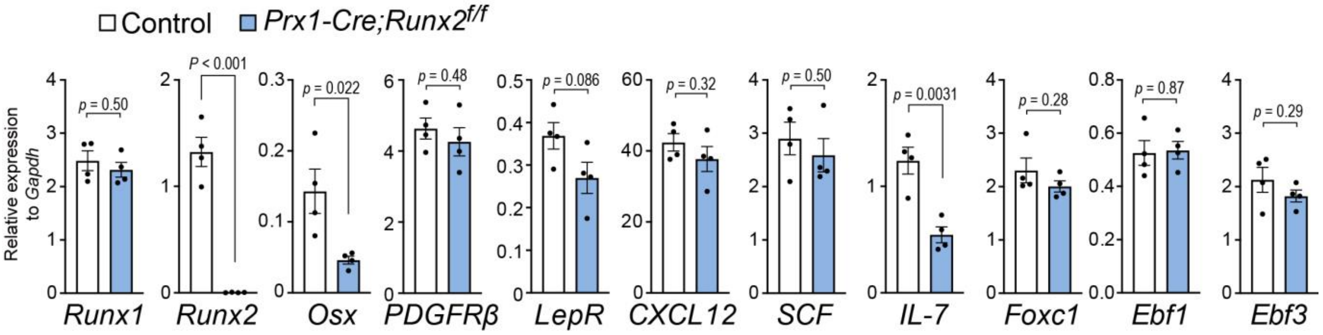


**Supplementary Figure 2. HSC niches are formed in mice lacking Runx2**

Relative mRNA expression levels of *Runx1*, *Runx2*, *Osx*, *PDGFRβ*, *LepR*, *CXCL12*, *SCF*, *IL-7*, *Foxc1*, *Ebf1*, and *Ebf3* in sorted CAR cells from 3-week-old control and *Prx1-Cre;Runx2*$^{f/f}$*;CXCL12-GFP* mice (n = 4 mice per group). All error bars represent SD of the mean. Statistical significances were calculated using the two-tailed unpaired Student's *t*-test. Source data are provided as a Source Data file.

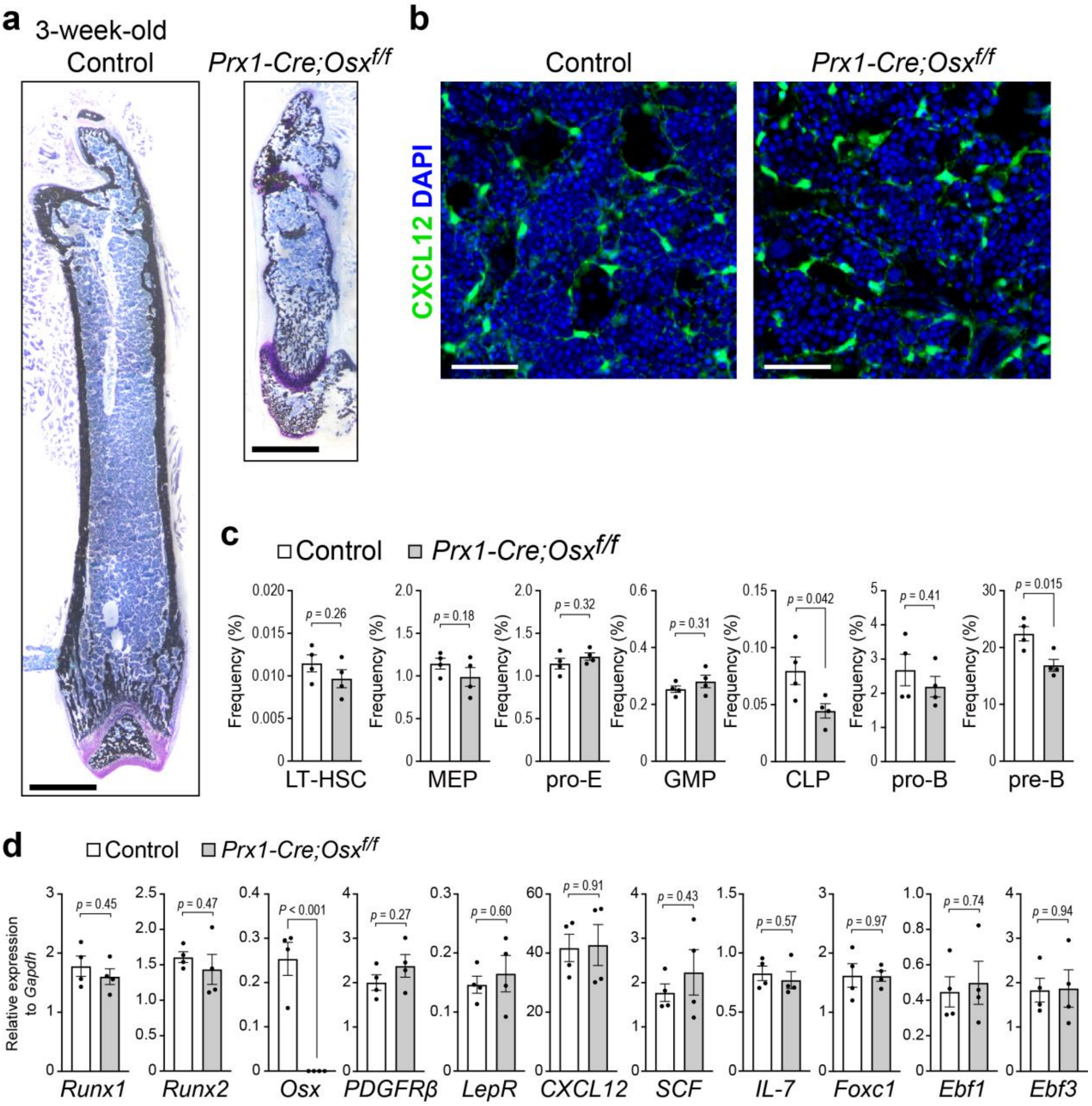


**Supplementary Figure 3. HSC niches are formed in mice lacking Osterix**

**a**-**d** Bone marrow from 3-week-old control and *Prx1-Cre;Osx*$^{f/f}$*;CXCL12-GFP* mice was analyzed.

**a** von Kossa and toluidine blue staining of femurs. Bars: 1 mm.

**b** Confocal microscopy images showing expression of CXCL12-GFP. Bars: 100 µm.

**c** Frequencies of LT-HSCs, MEPs, pro-E, GMPs, CLPs, pro-B cells, and pre-B cells (n = 4 mice per group).

**d** Relative mRNA expression levels of *Runx1*, *Runx2*, *Osx*, *PDGFRβ*, *LepR*, *CXCL12*, *SCF*, *IL-7*, *Foxc1*, *Ebf1*, and *Ebf3* in sorted CAR cells (n = 4 mice per group).

All error bars represent SD of the mean. Statistical significances were calculated using the two-tailed unpaired Student's *t*-test. Source data are provided as a Source Data file.

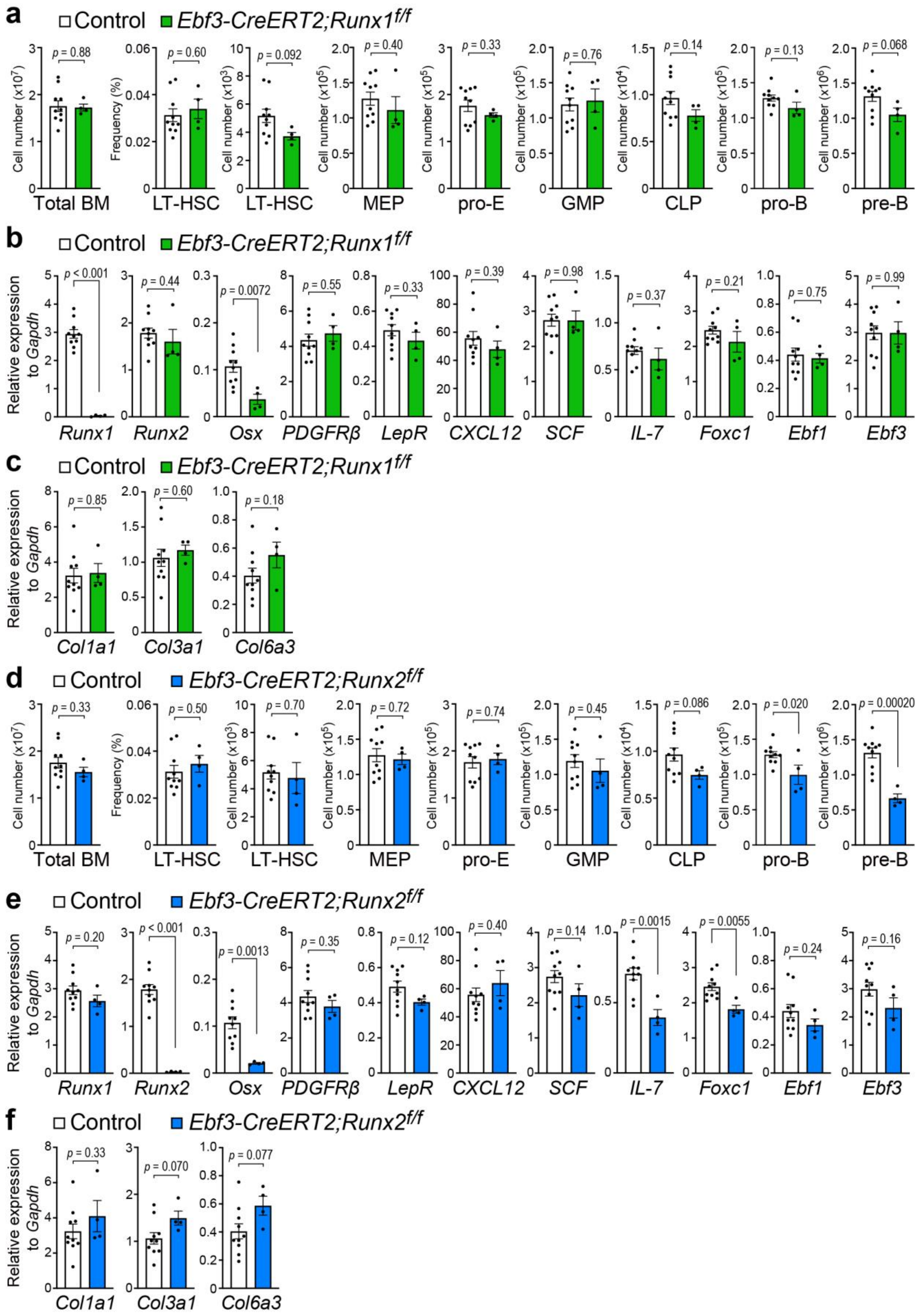

a
Control
Ebf3-CreERT2;Runx1f/f
Cell number (x10^7)
Frequency (%)
Cell number (x10^3)
Cell number (x10^5)
Cell number (x10^4)
Cell number (x10^6)
p = 0.88
p = 0.60
p = 0.092
p = 0.40
p = 0.33
p = 0.76
p = 0.14
p = 0.13
p = 0.068
Total BM
LT-HSC
LT-HSC
MEP
pro-E
GMP
CLP
pro-B
pre-B
b
Control
Ebf3-CreERT2;Runx1f/f
Relative expression to Gapdh
p < 0.001
p = 0.44
p = 0.0072
p = 0.55
p = 0.33
p = 0.39
p = 0.98
p = 0.37
p = 0.21
p = 0.75
p = 0.99
Runx1
Runx2
Osx
PDGFRβ
LepR
CXCL12
SCF
IL-7
Foxc1
Ebf1
Ebf3
c
Control
Ebf3-CreERT2;Runx1f/f
Relative expression to Gapdh
p = 0.85
p = 0.60
p = 0.18
Col1a1
Col3a1
Col6a3
d
Control
Ebf3-CreERT2;Runx2f/f
Cell number (x10^7)
Frequency (%)
Cell number (x10^3)
Cell number (x10^5)
Cell number (x10^4)
Cell number (x10^6)
p = 0.33
p = 0.50
p = 0.70
p = 0.72
p = 0.74
p = 0.45
p = 0.086
p = 0.020
p = 0.00020
Total BM
LT-HSC
LT-HSC
MEP
pro-E
GMP
CLP
pro-B
pre-B
e
Control
Ebf3-CreERT2;Runx2f/f
Relative expression to Gapdh
p = 0.20
p < 0.001
p = 0.0013
p = 0.35
p = 0.12
p = 0.40
p = 0.14
p = 0.0015
p = 0.0055
p = 0.24
p = 0.16
Runx1
Runx2
Osx
PDGFRβ
LepR
CXCL12
SCF
IL-7
Foxc1
Ebf1
Ebf3
f
Control
Ebf3-CreERT2;Runx2f/f
Relative expression to Gapdh
p = 0.33
p = 0.070
p = 0.077
Col1a1
Col3a1
Col6a3

**Supplementary Figure 4. Runx1 and Runx2 are dispensable for the maintenance of HSC niches**

**a**-**f** Bone marrow from 20- to 24-week-old tamoxifen-treated control (n = 10 mice), *Ebf3-CreERT2;Runx1*$^{f/f}$ (n = 4 mice) (**a-c**), or *Ebf3-CreERT2;Runx2*$^{f/f}$ (n = 4 mice) (**d-f**) mice was analyzed.

**a**,**d** Total hematopoietic cell counts, frequencies of LT-HSCs, and the numbers of LT-HSCs, MEPs, pro-E, GMPs, CLPs, pro-B cells, and pre-B cells in femurs and tibias.

**b**,**c**,**e**,**f** Relative mRNA expression levels of *Runx1*, *Runx2*, *Osx*, *PDGFRβ*, *LepR*, *CXCL12*, *SCF*, *IL-7*, *Foxc1*, *Ebf1*, and *Ebf3* (**b**,**e**), *Col1a1*, *Col3a1*, and *Col6a3* (**c**,**f**) in sorted CAR cells.

All error bars represent SD of the mean. Statistical significances were calculated using the two-tailed unpaired Student's *t*-test. Source data are provided as a Source Data file.

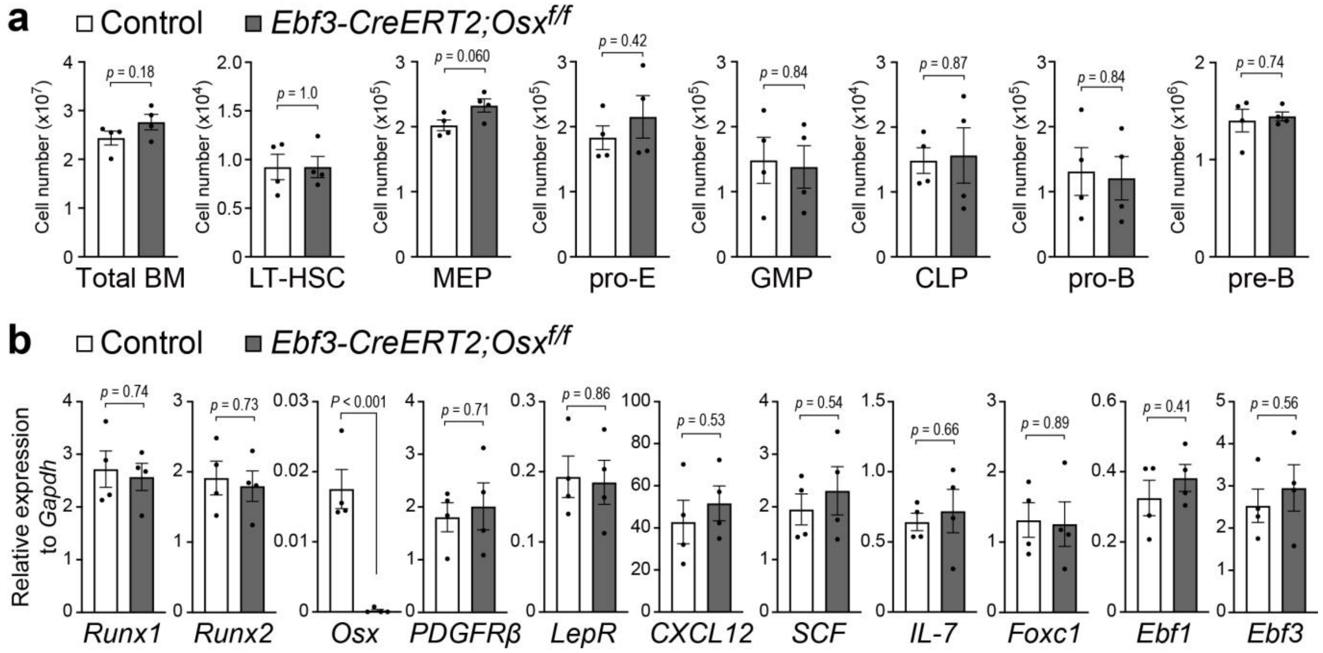


**Supplementary Figure 5. Osterix is dispensable for the maintenance of HSC niches**

**a**,**b** Bone marrow from 20- to 24-week-old tamoxifen-treated control and *Ebf3-CreERT2;Osx*$^{f/f}$ mice was analyzed.

**a** Total hematopoietic cell counts and the numbers of LT-HSCs, MEPs, pro-E, GMPs, CLPs, pro-B cells, and pre-B cells in femurs and tibias (n = 4 mice per group).

**b** Relative mRNA expression levels of *Runx1*, *Runx2*, *Osx*, *PDGFRβ*, *LepR*, *CXCL12*, *SCF*, *IL-7*, *Foxc1*, *Ebf1*, and *Ebf3* in sorted CAR cells (n = 4 mice per group).

All error bars represent SD of the mean. Statistical significances were calculated using the two-tailed unpaired Student's *t*-test. Source data are provided as a Source Data file.

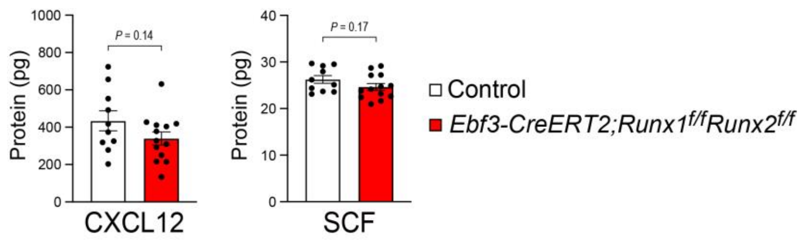


**Supplementary Figure 6. Protein expression of CXCL12 and SCF in mice lacking both Runx1 and Runx2 in CAR cells**

Humeri from 20- to 24-week-old tamoxifen-treated control (n = 10 mice) and *Ebf3-CreERT2;Runx1*$^{f/f}$*Runx2*$^{f/f}$ mice (n = 13 mice) were analyzed. All error bars represent SD of the mean. Statistical significances were calculated using the two-tailed unpaired Student's *t*-test. Source data are provided as a Source Data file.

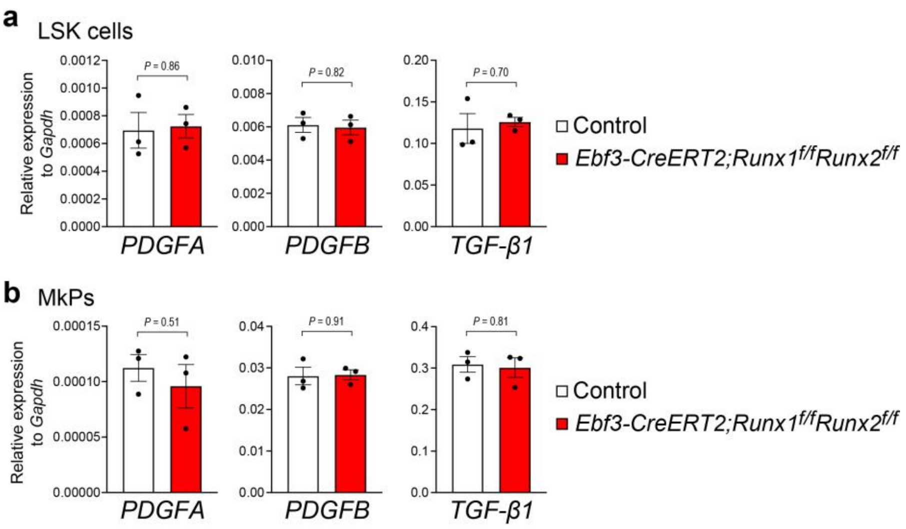


**Supplementary Figure 7. The expression of *PDGF*s and *TGF-β1* was unaltered in HSPCs and MkPs from mice lacking both Runx1 and Runx2 in CAR cells**

**a**,**b** Femurs and tibias from 20- to 24-week-old tamoxifen-treated control and *Ebf3-CreERT2;Runx1$^{f/f}$Runx2$^{f/f}$* mice were analyzed (n = 3 mice per group). Relative mRNA expression levels of *PDGFA*, *PDGFB,* and *TGF-β1* in sorted LSK cells (**a**) and MkPs (**b**). All error bars represent SD of the mean. Statistical significances were calculated using the two-tailed unpaired Student's *t*-test. Source data are provided as a Source Data file.

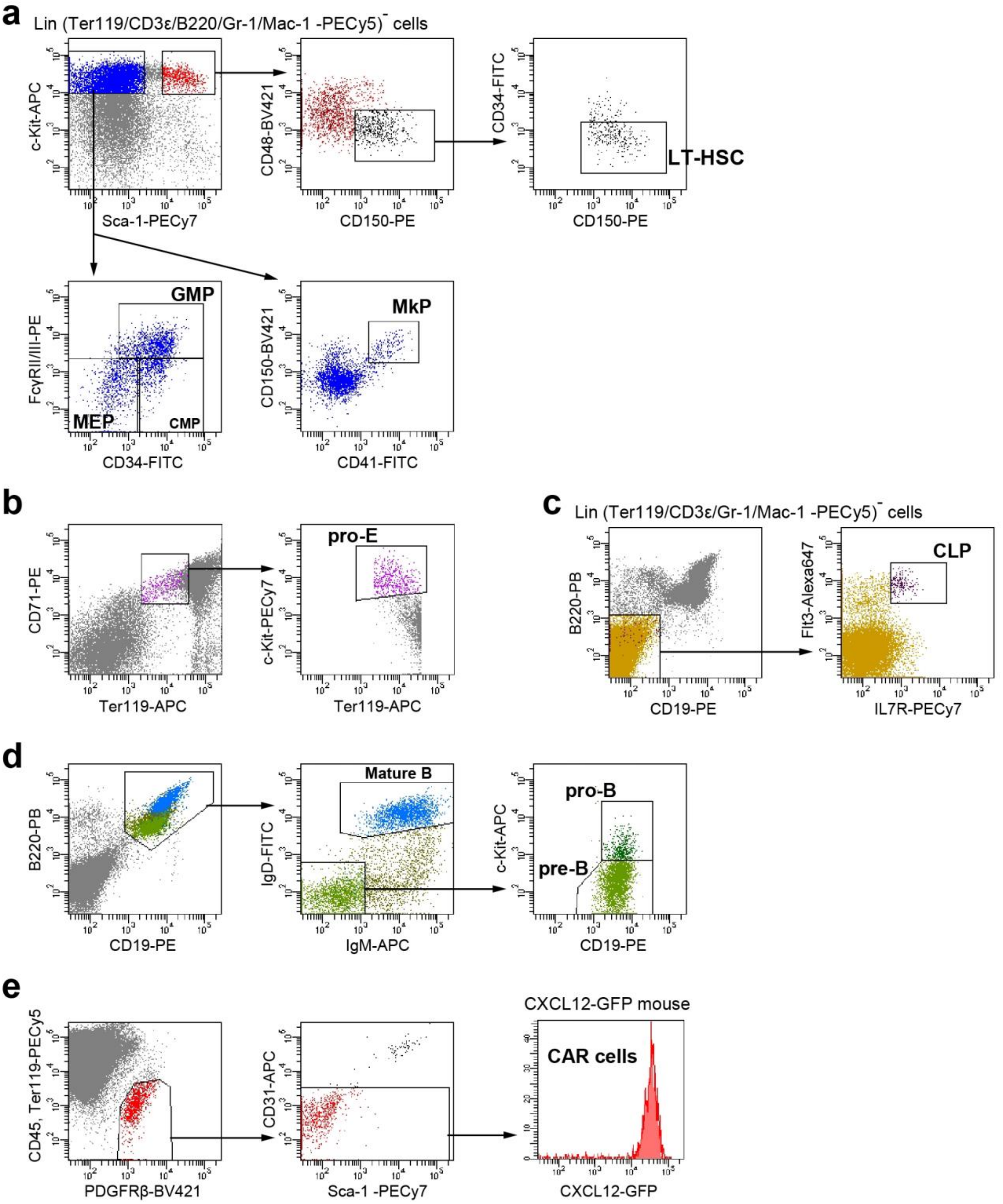


**Supplementary Figure 8. FACS Gating strategies for HSPCs and CAR cells**

FACS Gating strategies for LT-HSCs, MEPs, GMPs, Mkps (**a**), proerythroblasts (pro-E) (**b**), CLPs (**c**), pro-B cells, pre-B cells (**d**), and CAR cells (**e**).

| Gene Name | Sequence (5' - 3') |
|---|---|
| Mouse | |
| *Gapdh* | TCA TGA GCC CTT CCA CAA TG |
| | GGT GTG AAC CAC GAG AAA TAT GAC |
| *Runx1* | GGC AAC GAT GAA AAC TAC TCG GC |
| | TCT ACC GCT CCG CCC GAC |
| *Runx1b* (proximal) specific | ATG GGA ATT TTG CCT CCG GG |
| | GTG GGC AAC CAG AAT TCA AA |
| *Runx1c* (distal) specific | ATA GAA TCC CCC GCC TTC AG |
| | CTC TCA TGA AGC ACT GTG GA |
| *Runx2* | GAC TGT GGT TAC CGT CAT GGC |
| | GGG GAC CGT CCA CTG TCA C |
| *Osx (Sp7)* | ATG GCG TCC TCT CTG CTT G |
| | TGA AAG GTC AGC GTA TGG CTT |
| *Pdgfrb* | CAA CTC ACT AGG GCC GGA G |
| | GCA CGG AAT TGT CGT CTC AG |
| *Lepr* | ATG CCC CAA TTT CAA ACC TG |
| | GGA ACC TTG AGG CTT CTT GGA |
| *CXCL12* | CCA GAG CCA ACG TCA AGC AT |
| | CAG CCG TGC AAC AAT CTG AA |
| *SCF (Kitl)* | GGT AGC TAG TTC TAT CCA TGC GGT |
| | CCT GTA AGG ACT TTT CTG GAG AGT CT |
| *IL7* | TCC TCC ACT GAT CCT TGT TC |
| | CTT CAA CTT GCG AGC AGC AC |
| *Foxc1* | GGT ACA GAG ACT GAC TGG CA |
| | TTC TAA CCT GCG GAA ATC CAAC |
| *Ebf1* | CAC TCG GTG CGG GAA ATG T |
| | CAC AAA GGC CGT CCT CTC AA |
| *Ebf3* | CGA AAG GAC CGC TTT TGT GG |
| | AGT GAA TGC CGT TGT TGG TTT |
| *Col1a1* | CAC CCT CAA GAG CCT GAG TC |
| | GTT CGG GCT GAT GTA CCA GT |
| *Col3a1* | CTG TAA CAT GGA AAC TGG GGA AA |
| | CCA TAG CTG AAC TGA AAA CCA CC |
| *Col6a3* | GAG GTT GGA AAT TGG ACA GGA C |
| | CCG TAC AGC AGT TAT GGC ATC |
| *Pdpn* | GAG GGC TTA ATG AAT CTA CTG GC |
| | GAC AGT TCC TCT AAG GGA GGC |
| *Pdgfra* | CCC TAT CCT GGC ATG ATG GTC |
| | TGA GGT GGT AGA AGG AGG GTC |
| *Gli1* | CCA AGC CAA CTT TAT GTC AGG G |
| | AGC CCG CTT CTT TGT TAA TTT GA |
| *Ly6a* | AGG AGG CAG CAG TTA TTG TGG |
| | CGT TGA CCT TAG TAC CCA GGA |
| *Pdgfa* | TGG CTC GAA GTC AGA TCC ACA |
| | TTC TCG GGC ACA TGG TTA ATG |
| *Pdgfb* | AAG TGT GAG ACA ATA GTG ACC CC |
| | CAT GGG TGT GCT TAA ACT TTC G |
| *Tgfb1* | CTT CAA TAC GTC AGA CAT TCG GG |
| | GTA ACG CCA GGA ATT GTT GCT A |

**Supplementary Table 1. The primer pairs used for qRT-PCR**
